# Repetitive DNA profile of the amphibian mitogenome

**DOI:** 10.1186/s12859-020-3532-8

**Published:** 2020-05-19

**Authors:** Noel Cabañas, Arturo Becerra, David Romero, Tzipe Govezensky, Jesús Javier Espinosa-Aguirre, Rafael Camacho-Carranza

**Affiliations:** 1 Instituto de Investigaciones Biomédicas, Universidad Nacional Autónoma de México, Cd. Universitaria, 04510, Cd. Mx., Mexico; 2 Facultad de Ciencias, Universidad Nacional Autónoma de México, Cd. Universitaria, 04510, Cd. Mx., Mexico; 3 Centro de Ciencias Genómicas, Universidad Nacional Autónoma de México, Cuernavaca, Morelos Mexico

**Keywords:** Direct and inverted repeat DNA sequences, mtDNA, Mitogenome, Amphibians

## Abstract

**Background:**

Repetitive DNA elements such as direct and inverted repeat sequences are present in every genome, playing numerous biological roles. In amphibians, the functions and effects of the repeat sequences have not been extensively explored. We consider that the data of mitochondrial genomes in the NCBI database are a valuable alternative to generate a better understanding of the molecular dynamic of the repeat sequences in the amphibians.

**Results:**

This work presents the development of a strategy to identify and quantify the total amount of repeat sequences with lengths from 5 to 30 base pairs in the amphibian mitogenomes. The results show differences in the abundance of repeat sequences among amphibians and bias to specific genomic regions that are not easily associated with the classical amphibian ancestry.

**Conclusions:**

Derived from these analyses, we show that great variability of the repeat sequences exists among amphibians, demonstrating that the mitogenomes of these organisms are dynamic.

## Background

Amphibians are a class of ectotherms organisms widely distributed over the planet, cataloged as mediators and biosensors of the equilibrium of ecosystems [1, 2]. Thus, it is alarming that more than 70% of the amphibian species suffer a population decline due to a multi-causal phenomenon, which includes reproductive failures, predation, pathogens, pollution, and destruction of their habitats [2–4]. Therefore, it is necessary to expand our understanding of the biological phenomena linked to these organisms and contribute to mitigating the possible effects caused by their decline [5, 6].

The amphibian mitochondrial genome (mitogenome) is a valuable source of information that represents an alternative for evolutionary and taxonomic studies when there is a shortage of nuclear genome data in public databases, this includes fully sequenced amphibian genomes and high-quality genome assemblies [7–9]. The mitogenome is constituted by a multi-copy circular DNA molecule of ~ 17.5 Kbp in size that encodes ~ 37 genes and maintains a stable structure with changes caused only by point mutations [10–13]. Nonetheless, there is increasing evidence of mitochondrial genomes that present remarkable structural changes such as repeat DNA sequences, gene duplications, and several other genomic rearrangements [14–17].

**Fig. 1:**
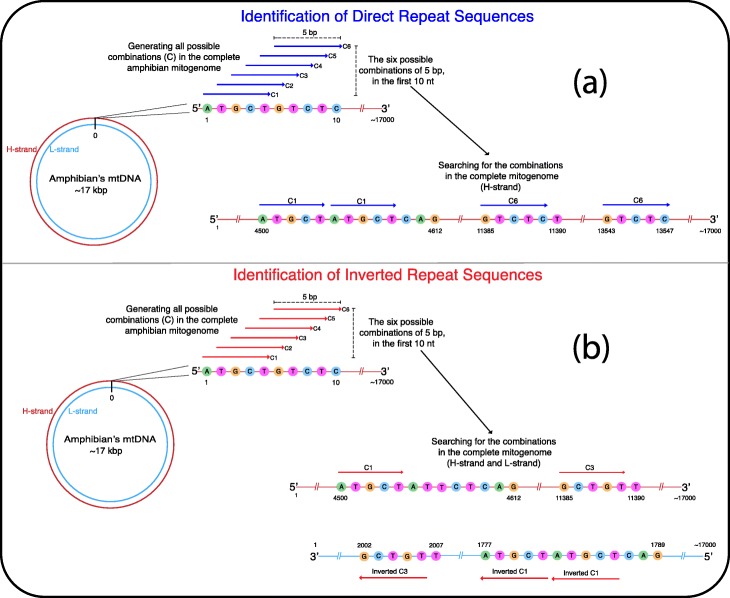
The strategy implemented to identify the direct and inverted repeats. **a** The figure presents examples of the identification of direct repeats of 5 bp. The strategy firstly consisted in generating every possible combination of 5 bp that can be present in the DNA sequence of the amphibian mitogenome, and secondly performing the search of these combinations in the DNA chain 5′➔3′ to identify if they are repeated in the mitogenome. **b** The strategy to identify the inverted repeats consisted in generating all the possible combinations of DNA sequences with a length of 5 bp and performing the search of these sequences in an inverted way in the DNA chain 3′➔ 5′. This strategy was implemented to identify repeat sequences with lengths from 5 to 30 bp

The repeat DNA sequences (repeats) are especially important because they take part in numerous biological processes as sequence motifs or modelers of the genomic structure [18, 19]. Many classifications of them have been proposed; they are nevertheless divided into two main types: Direct Repeat Sequences (DRs) and Inverted Repeat Sequences (IRs). They are both DNA sequences present more than once in a genome, but the characteristic of the direct repeats is that they are located on the same strand, in the same direction. In contrast, the inverted repeats are found on any of the two DNA strands, but we localize the repeat sequences on the complementary DNA strand in an inverted orientation (Fig. 1) [20–23].

Repeats in eukaryotes serve as promoters, enhancers, or silencers during DNA replication, they regulate the formation of the chromatin or produce repetitive domains of proteins [18, 20–22]. In prokaryotes, repeat sequences participate in a system of defense against foreign DNA called CRISPR, and during the processes of DNA replication, repair, and recombination, direct and inverted repeats are primary sources for the generation of secondary structures or non-B DNA conformations such as triplex or slipped structures which can result in genomic rearrangements. For example, the direct repeats can form stem-loops, or two pairs of inverted repeats can generate cruciform structures, which can lead to deletions, duplications, or inversions depending on the mechanisms involved [20, 24–28].

Regarding the repeats in mitochondrial genomes, they contribute to the generation of phenotypes in lifespan, heart failures, or pathologies such as cancer or Parkinson’s disease, but their roles others than these effects are poorly understood [6, 29–33]. For example, it is possible to encounter repeats in the control region of the mtDNA of vertebrates, although many of them with no known function. Repeats are as well associated with frequent mitochondrial rearrangements such as deletions, duplications, translocations, and more rarely inversions, which affect the order or copies of genes such as mitochondrial tRNAs in birds, reptiles, fish, and amphibians. A model named tandem duplication-random loss has been suggested as a mechanism to explain some of these rearrangements, but there are cases without a plausible explanation proposed [14–17, 32, 34].

Numerous classifications, algorithms, and strategies have been explored and proposed to study the vast diversity of repeats that exist [23, 26, 28, 29, 31, 35]. According to Repbase and Dfam, the reference databases for repeat sequences, the efforts are focused on the identification of eukaryotic repetitive sequences such as transposable elements, simple repeats, or gene duplications [36, 37]. While in mitochondria, the repeats databases ChloroMitoSSRDB and MitoSatPlant are mainly linked to the study of microsatellites [38, 39]. Some examples of tools employed for the identification of repeat sequences are RepeatMasker based in a library of repeats and search by homology; REPET and PRAP, which are based in algorithms of local pairwise alignment; RepeatFinder, REPuter, and Repeat-match which use a sequence seed to localize perfect and degenerated repeats (For more details about the tools and strategies to identify repeats see the review of Lerat, 2010) [23, 35].

Only a few comprehensive studies have searched for repeat sequences in mitochondrial genomes. First, Goios et al. 2006 examined the human mitogenomes to identify the direct and inverted repeat sequences [40]. Second, the study of Čechová et al. 2018, which identified variability in the number of mitochondrial inverted repeat sequences in plants and fungi compared with mammals [41]. Third, the database and software developed by Shamanskiy et al. 2019 to identify degenerated direct and inverted repeat sequences in mitochondrial DNA [23]. Last but not least, the investigation of Yang et al. 2013 that performed the identification of repeats and reported a correlation of the inverted repeats and lifespan of mammals and additionally presented experiments that suggest an accumulation of inversions related to age mediated by inverted repeats in mouse [29]. In comparison with the aforementioned studies, in this work, we propose a comprehensive strategy to study all the possible direct and inverted repeat sequences in the amphibian mitochondria. We cover the study of coding and no-coding regions and focus our analyses in the variability of repeat sequences and their possible evolutionary value rather than solely the identification.

Therefore, our objective was to identify, quantify, and compare the repeat sequences in mitochondrial genomes, widespread organelles among almost all eukaryotes. We focused on the amphibians, which present a great diversity of adaptations and evolutionary histories [9, 10, 15].

## Results

### Identification of direct and inverted repeat sequences

We considered that any direct and inverted repeat sequence is a potential substrate for the generation of genomic changes; therefore, they must be subject to selection pressures that mark their distribution, abundance, and size, reflecting their evolutionary histories. Using this as a fundament, we searched, in the complete mtDNA sequence of every amphibian, all combinations of DNA sequences with lengths from 5 to 30 bp and 100% identity. As an example (Fig. 1), a script first opens a file that contains a complete mitogenome sequence, and starting from the first nucleotide generates strings combinations of size five advancing one by one nucleotide, which results in overlapped sequences that are temporarily stored in a list. Once the list is ready, the redundant combinations are eliminated. Secondly, the script scans the complete mitogenome in a 5′➔3′ direction seeking for the exact position and the number of copies of each five nucleotides string. We designated these sequences as direct repeat sequences. Subsequently, the script uses the mitogenome sequence to create the complementary DNA sequence (3′➔5′) and then invert it to a 5′➔3′ direction. The sequence is now set to identify the inverted repeat sequences; thus, the script searches the combinations saved in the initial list, but in the inverted mitogenome sequence to localize the exact position and the number of repetitions. The process is iterated to identify the repeat sequences of 6, 7, 8, 9, 10, to 30 nucleotides, and the data collected are saved in file texts every time.

### Examining the abundance of repeat sequences in the amphibians

**Fig. 2:**
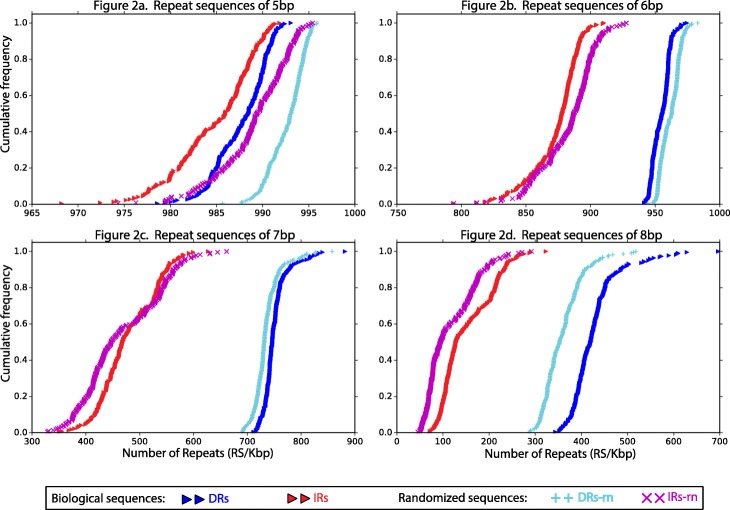
Empirical distributions of the abundance of repeat sequences with lengths from 5 to 8 bp. The abundance of direct and inverted repeats in the 221 mitogenomes, and their comparison with the abundance of repeats in randomized DNA sequences, each figure is an independent analysis for a specific size of repeat sequences. Each point in the distributions represent an organism and the average number of repeats per Kbp. **a** Empirical distributions of repeat sequences of 5 bp, **b** Empirical distributions of repeat sequences of 6 bp, **c** Empirical distributions of repeat sequences of 7 bp and (**d**) Empirical distributions of repeat sequences of 8 bp. To review the distributions of sizes from 5 to 20 bp go to Additional file 2. (Abbreviations used in the figure are interpreted as follows **DRs**: Direct Repeat sequences; **IRs**: Inverted Repeat sequences; **DRs-rn**: Random Direct Repeat Sequences; **IRs-rn**: Random Inverted Repeat Sequences; **RS/Kbp**: Number of repeat sequences divided by the mitogenome size in kilobase pairs)

The empirical distributions show that overall the direct repeats are always more abundant than the inverted repeats (*P* < 0.01), and for both, abundance decreases as repeats size increases (Fig. 2). The distributions additionally present, as a control, the comparisons of the results of the biological sequences (mitogenomes) versus the results obtained from randomized sequences that maintained the same GC content and size as the mitogenomes sequences to ensure valid comparisons (see Statistical analyses in Materials and methods). The biological sequences versus the randomized sequences display the same tendencies, but interestingly the randomized direct and inverted repeats of 5 bp and 6 bp show more abundance of repeats than biological sequences (*P* < 0.01) (Fig. 2a and b); for direct repeats of 7 bp (Fig. 2c) biological sequences show more abundance of repeats than randomized ones (*P* < 0.01), but for inverted repeats, there is no significant difference between biological and randomized sequences; for repeat sequences of 8 bp (Fig. 2d) biological sequences are more abundant than randomized ones (*P* < 0.01). For repeats greater than 8 bp random sequences are less abundant than biological ones; in fact, inverted repeats of 14 bp or more, and direct repeats of 20 bp or more were not observed in the randomized sequences. In the biological sequences, the inverted repeats of 24 bp or more were not observed, but direct repeats of 30 bp are present in 62% of the species studied (Additional file 2). Empirical distributions of direct repeats of 9 bp or more are asymmetric, showing some species with few direct repeats and some others with many direct repeats, which means more variability among species.

Variability among species increases as the size of the repeat sequences increase. Inverted repeats present more variability than the direct repeats (*P* < 0.01), except for repeats of 8 bp, where variability among species is not statistically different. For repeat sequences greater than 8 bp, the direct repeats have more variability than the inverted ones. When comparing biological sequences with random ones from 5 bp to 8 bp, the variability among species obtained is generally similar for both biological and randomized sequences (Fig. 2), while for larger repeats the variability is smaller in random sequences than in biological ones (Additional file 2).

### The behavior of the abundance of repeat sequences in the amphibian phylogenetic tree

**Fig. 3:**
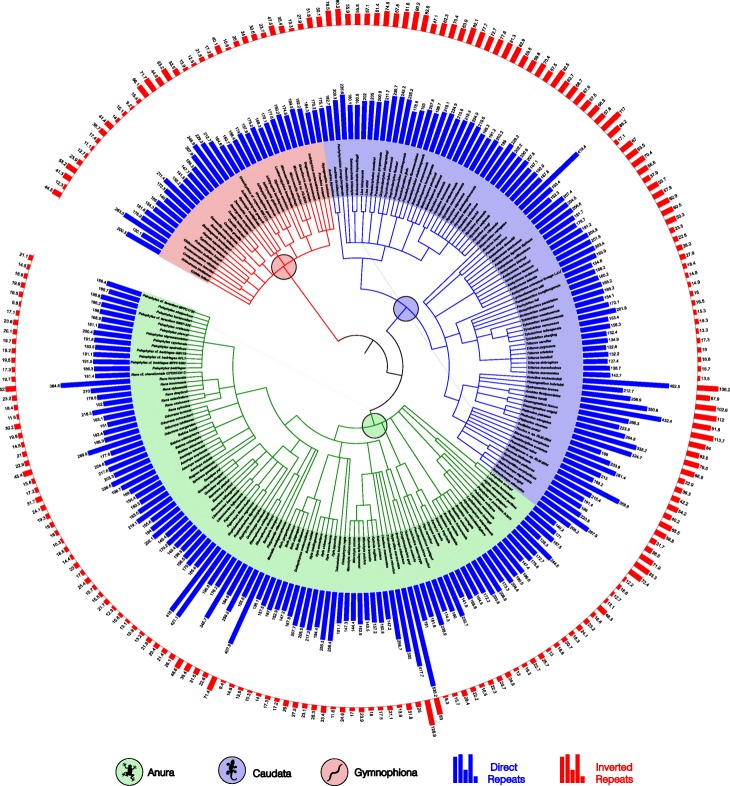
The abundance of repeat sequences of 9 bp in the amphibian phylogenetic tree. The figure presents the 221 amphibian mitogenomes classified into three orders, with their respective abundance of direct and inverted repeat sequences shown as bar graphs. The amphibians with a higher abundance of direct repeats seem to appear as random events in the amphibian phylogenetic tree, and the same pattern is observed for the inverted repeats of the orders Anura and Gymnophiona. On the contrary, the majority of the amphibians of the order Caudata display an increase abundance of inverted repeats. The number above each bar graph represents the abundance of repeat sequences (repeats /Kbp). The images with distributions of repeat sequences of other lengths can be accessed from Additional file 3

Using the accepted phylogenetic amphibian classification of three orders (Anura, Caudata, and Gymnophiona), we identified that among the amphibian mitogenomes there are variations in the abundance of repeat sequences, which are characteristic for each size and type of repeat (direct or inverted). Figure 3 shows the example of repeat sequences of 9 bp in the 221 amphibians, which present an average abundance of ~ 180 direct repeats per Kbp, and several cases of organisms with atypical abundances of direct repeats such as *Breviceps adspersus* (~ 535 repeats /Kbp) of the order Anura, *Ensatina eschscholtzii* (~ 452 repeats/Kbp) of the order Caudata, or *Crotaphatrema lamottei* (~ 263 repeats/Kbp) of the order Gymnophiona. In the case of the inverted repeats of 9 bp, the average abundance is ~ 24 inverted repeats per Kbp, and there atypical cases with higher abundance too, such as *Hyperolius marmoratus* (~ 126 repeats /Kbp) of the order Anura, *Rhyacotriton variegatus* (117 repeats /Kbp) of the order Caudata, or *Dermophis mexicanus* (~ 71 repeats /Kbp) of the order Gymnophiona, but the differences in the abundances of inverted repeats are less drastic compared with the direct repeats.

The amphibians that exhibit a greater abundance of direct repeats do not always coincide with an increase in the abundance of inverted sequences. The increase of direct repeats seems to emerge as random events in the organisms of the three amphibian orders, and the same pattern is observed for the inverted repeats of the orders Anura and Gymnophiona. Conversely, the majority of the amphibians of the order Caudata display an increase abundance of inverted repeats (Fig. 3). To see the differences in the abundance of repeat sequences of other sizes go to Additional file 3.

### Mapping the repeat sequences in the amphibian mitogenome

**Fig. 4:**
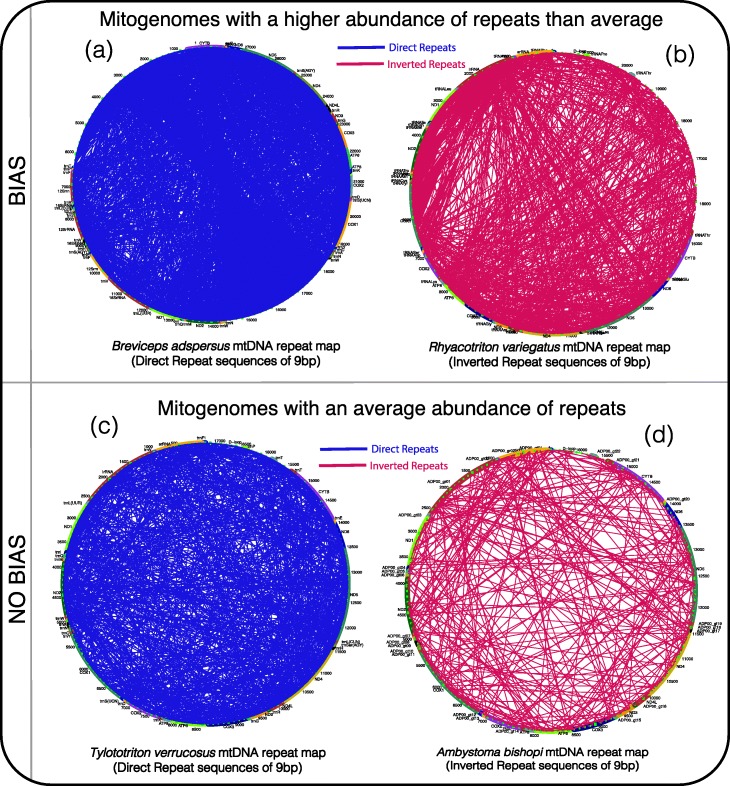
Distribution of the repeat sequences in the amphibian mitogenomes. The amphibian mitogenomes with an atypical abundance of repeats show a preference for particular genomic regions, while the repeats in the mitogenomes with an average abundance of repeats are distributed all over the genome without preference. **a** The distribution of direct repeats of 9 bp in the mitogenome of *Breviceps adspersus* has a bias towards non-genic regions, principally to the D-Loop region. **b** The distribution of the inverted repeats of 9 bp in the mitogenome of *Breviceps adspersus* shows the bias towards genic regions. On the contrary, the direct repeats of (**c**) *Tylototriton verrucosus* and the inverted repeats of (**d**) *Ambystoma bishop* do not exhibit a preference for any genomic region

The analyses implemented for the identification of direct and inverted repeats allowed us to encounter these sequences throughout the entire amphibian mitogenomes. Nevertheless, we observed that the amphibians that present a greater abundance repeats than the average display biases of the repeats in particular genomic regions. The mitogenomes with this atypical abundance of repeats have a bias of the distribution of the direct repeats in non-genic regions, while the inverted repeats are mainly encountered in genic regions. Figure 4a illustrates the case of *Breviceps adspersus,* an amphibian with an increased abundance of direct repeats than the average, that shows that the direct repeats of 9 bp are principally present in non-genic regions. The bias can be spotted when comparing against a mitogenome that possesses an average abundance of repeats, for example, *Tylototriton verrucosus* that does not exhibit a bias towards a specific mitochondrial region; instead, this amphibian exposes a distribution of the direct repeats all over the mitogenome (Fig. 4c). This bias is clearer when we compare the distribution of the direct repeats of 30 bp that are less abundant (Additional file 4). On the other hand, Fig. 4b shows that mitogenomes, with an atypical abundance of inverted repeat sequences of 9 bp than the average, present bias to genetic regions as the map of the inverted repeats of *Rhyacotriton variegatus* depicts*.* While the repeat map of *Ambystoma bishopi*, an amphibian with an average abundance of inverted repeats, displays these sequences all over the mitogenome, with no evident preference for a genomic region (Fig. 4d). To see more examples of mapping of the repeat sequences go Additional file 4.

## Discussion

The identification of all the repeat sequences in the mitogenomes is a suitable way to obtain an overview of the repeats with less bias since they are likely to have many unidentified functions, and the forces that regulate their dynamics are also unknown. Therefore, our approach is based on the premise that every repeat sequence could be a potential source for the formation of genomic rearrangements. However, we need to broaden the explication and justification of certain parameters and comparisons implemented in the strategy in order to obtain a better understanding of the results previously presented. First, the applied strategy generated redundancies since we took into account overlapped repeats, sequences that form part of other sequences partially, as independent findings (Fig. 1). Nonetheless, we considered that the overlapped repeats should be included when identifying repeats since they are a significant source for the formation of slipped-strand DNA structures during the replication or reparation of the DNA, a phenomenon probably essential for the mtDNA since recombination is not frequent in these genomes. Second, the literature reports the identification of repeat sequences with sizes between 5 and 25 bp in mitochondrial genomes, the reason for which we opted for the identification of repeats with lengths from 5 bp to 30 bp. According to our results, it proved to be an adequate range to identify almost all repeats in mitogenomes, but our strategy can be implemented to identify larger repeat sequences [26, 29, 31, 32, 41]. Third, we sought for sequences with 100% identity, which might seem to be a parameter that decreases the sensitivity of our method to identify degenerated repeats, which probably are frequent in the mitogenomes due to their high rates of point mutations. However, our strategy is still able to detect degenerated repeat sequences due to the identification of overlapped repeats [6, 42, 43]. Lastly, once we identified the repeats in the mitogenomes, we searched the repeats in random sequences that maintained the GC content and the genome size. The identification of repeats in the biological sequences and random sequences permitted us to make comparisons that proved that their abundances differ from each other (Fig. 2), which probably emphasizes the effect of biological processes involved in the generation of the repeats.

Regarding the results, they provide a general description of the repeats in the amphibian mitogenomes and show some unexpected characteristics in their behavior worthwhile to discuss. To begin with, direct and inverted repeats of small lengths (5 to 10 bp) are abundant (Fig. 2), but the abundance of larger repeats (> 15 bp) seems to decline drastically as their size increase, for which is suitable to think that their generation is under restrictions due to the mitogenome size. These results also fit with the current model that posits that recombination rates in the mitogenomes are low, but rates of replication are higher; and with the proposal that replicative mechanisms are more likely to use small repeat sequences to produce genomic changes [26, 31, 43].

The direct repeats are more abundant than the inverted repeats. We suggest that it is due to two main reasons. Firstly, the mechanisms involved in their generation. Direct repeats are able to emerge using as a template a single DNA strand, but the formation of inverted repeats needs both strands, which convert them in events energetically more demanding [20, 26]. Secondly, the different roles that the repeats play in the mitogenomes. Inverted sequences are found in less abundance compared with the direct repeats because they probably are able to generate genetic changes that could be fixed, such as inversion rearrangements that can drastically affect the structure and integrity of the mitogenome. In contrast, the direct repeats are more abundant since they permit the generation of variability in the mitogenomes by means of deletions and duplications mainly in the mitochondrial control region, where changes with no lethal effects are generated as the tandem duplication-random loss model suggest [9, 15, 32, 34, 43, 44].

The order Anura presents a higher abundance of repeats compared with the orders Caudata and Gymnophiona. This can be the result of bias in the dataset analyzed considering that 46% of the 221 mitogenomes belong to the order Anura, which increased the probability of finding the specimens that have a greater number of repeat sequences in this order. Additionally, we were aware that mitogenomes shared an evolutionary history, which can introduce a bias in the results due to phylogenetic covariance. Thus, to avoid that the degrees of freedom of our statistical tests may be inflated, we calculated the tests using half the original degrees of freedom and found that differences reported remain statistically significant (See *Statistical analyses*).

Amphibians with an atypical abundance of repeats present biases to specific genomic regions. Former studies have reported that repeats are mainly found in the D-Loop region and regulatory regions [33, 41]. We were not able to identify a preference of the repeats for some genomic region (Fig. 4c and d), except in the mitogenomes with an atypical abundance of repeats, where we identified that the direct repeats are mostly distributed in non-genic regions and the inverted repeats in genic regions (Fig. 4a and b). The amphibians that exhibit a greater abundance of direct repeats do not always coincide with an increase in the abundance of inverted sequences, which suggests that the two types of sequences are differentially regulated. Direct repeats are more dynamic and able to produce no lethal genomic changes in non-genic regions, and inverted repeats are less frequent and able to cause structural changes that could affect the integrity or gene expression of a mitogenome. We additionally suggest that the repeats sequences are dynamic and are constantly shaping the mitogenomes at a high frequency, which leads to encounter amphibians with atypical abundances of repeats.

Against the idea that mitogenomes have a stable structure because they are mainly coding DNA, we assess that it is an assertion based on studies of organisms with low genetic variability and recent evolutionary histories. In this work, we selected the amphibians as a study model. They are an ancient lineage that represents the transition between the aquatic and terrestrial vertebrates, have a widespread global distribution, and present distinctive phenotypes, characteristics that suggest multiple adaptations and evolutionary histories [10, 42, 44]. We were expecting that the repeats followed a pattern of change similar to the evolution of amphibians, but the comparisons of the repeats abundance did not seem to follow this pattern. Instead, we realized that the variability and dynamics of the abundance of repeats are greater than expected.

Based on the mitogenomic plasticity identified in the amphibians, we suggest that the abundance of repeat sequences, the type (directed or inverted), the distributions in the mitogenome, and the length of the repeats can be used to build profiles of repeat sequences as a potential tool to help in the identification and classification of amphibians, but we need more development of this profiles. This strategy is not limited to a particular group of organisms but can be implemented in any mitogenome (Additional file 5).

## Conclusions

We identified all the direct and inverted repeat sequences in the amphibian mitogenomes and demonstrated the presence of variability in the abundance of repeat sequences among the amphibians. We also detected that the amphibians with an atypical abundance of repeats present bias of the direct repeats towards non-genic regions, and of inverted repeats towards genic regions. Finally, we present the profiles of repeats sequences of the amphibian mitogenome based on abundance, type, distribution, and comparisons of repeats.

## Methods

### Data collection

We collected 221 sequences of amphibian mitochondrial genomes and their biological data from The National Center for Biotechnology Information (NCBI: www.ncbi.nlm.nih.gov/). Incomplete sequences or sequences with obsolete access numbers were excluded from the analyses. In some cases, we verified or completed the information with data from AmphibiaWeb (amphibiaweb.org) and relevant publications. This information is available in Additional file 1.

### Data classification

The amphibian mitogenome sequences were classified in three orders following the accepted phylogenetic classification from NCBI taxonomy (www.ncbi.nlm.nih.gov/taxonomy) and Amphibiaweb: Anura (*n* = 102), Caudata (*n* = 86), and Gymnophiona (*n* = 33) (Additional file 1). This classification was displayed as phylogenetic trees generated in iTOL: Interactive Tree Of Life V.3. (itol.embl.de/) [45].

### Identification of the repeat sequences on the amphibian mitogenomes

We developed Python scripts to identify every direct repeat and inverted repeat sequence with lengths from 5 to 30 base pairs (bp) and 100% identity in each one of the 221 amphibian mitochondrial genomes included in the analyses. The strategy is displayed in Fig. 1**,** and the scripts and detailed information can be accessed from the GitHub link in Availability of data and materials.

### Statistical analyses

In order to compare biological sequences with random ones with the same GC content and genome size, each mitogenome sequence was randomized 10 times using Python’s random module. Random sequences were analyzed in the same way as biological ones as a control.

To illustrate the distribution of the abundance of different sizes of repeat sequences in the 221 amphibian mitogenomes, we used empirical distributions, which are cumulative distributions associated with the empirical results of a sample. In this study, its value at a given point is equal to the proportion of observations from the sample whose abundance is less than or equal to that point. The variable used to build the empirical distributions was the abundance of repeat sequences (repeats /Kbp) calculated as the quotient between the number of repeat sequences and genome size in kilobase pairs (Kbp).

ANOVA was used to test the statistical significance of differences among direct repeats, inverted repeats, random direct repeats (DRs-rn), and random inverted repeats (IRs-rn). Levene test was used to test variance homogeneity. Since variances were not homogenous, the Welch test was done. Moreover, when one works with species that do not represent statistically independent data-points, degrees of freedom may be inflated, thus we calculated the ANOVA and post-hoc tests using half the original degrees of freedom, and found that differences reported remain statistically significant.

### Mapping of the direct repeat sequences and inverted repeat sequences

The localization of the repeat sequences in the mitogenomes was depicted by maps created with a script in R programming provided by Jiang-Nan Yang from the Leibniz Institute on Aging [29]. The program use a GenBank file to specify the type of sequence (circular for amphibian mitogenomes), the name of the genes and their exact positions in the mitogenome. Subsequently, the script read a file that contain the repeat sequences previously identified and the exact positions, these information is used to draw lines as representations of the positions of the mitochondrial repeat sequences.

## Supplementary information

**Additional file 1: Table S1.** Dataset of the amphibian mitogenomes analyzed.

**Additional file 2: Figure S2.** Empirical distributions of the Abundance of repeat sequences with lengths from 5 to 20 bp.

**Additional file 3: Figure S3.** The Abundance of repeat sequences of 9 bp, 7 bp, 9 bp,15 bp in the amphibian phylogenetic tree.

**Additional file 4: Figure S4.** Bias of the direct and inverted repeat sequences in the amphibian mitogenomes with an atypical abundance of repeats.

**Additional file 5.** Examples of the possible uses of the repeat sequences for identification and classification of amphibians.

## Data Availability

The authors declare that the data supporting the findings of this study are available within the main manuscript and Additional files. The datasets analyzed during the current study, the scripts used for the analyses, and the Additional files are available online at: https://github.com/SalmonellaIIB/Repeat_profiles.git

## Abbreviations

NCBI: National Center for Biotechnology Information
Mitogenome: Mitochondrial genome
mtDNA: mitochondrial DNA
Repeats: Repeat DNA sequences
DRs: Direct Repeat Sequences
IRs: Inverted Repeat Sequences
DRs-rn: Random Direct Repeat Sequences
IRs-rn: Random Inverted Repeat Sequences
RS/Kbp: Number of repeat sequences divided by the genome size in kilobase pairs
Kbp: Kilobase pairs
bp: base pairs
tRNAs: transfer RNA

## References

[1] Zardoya R, Meyer A (2001). On the origin of and phylogenetic relationships among living amphibians. Proc Natl Acad Sci U S A.

[2] AmphibiaWeb: Information on amphibian biology and conservation <https://amphibiaweb.org> University of California, Berkeley, CA, USA. Accessed 9 Apr 2019.

[3] Vences M, Thomas M, Bonett RM, Vieites DR (2005). Deciphering amphibian diversity through DNA barcoding: chances and challenges. Philos Trans R Soc Lond B Biol Sci.

[4] Hayes TB, Falso P, Gallipeau S, Stice M (2010). The cause of global amphibian declines: a developmental endocrinologist's perspective. J Exp Biol.

[5] Stuart SN, Chanson JS, Cox NA, Young BE, Rodrigues AS, Fischman DL, Waller RW (2004). Status and trends of amphibian declines and extinctions worldwide. Science.

[6] Alexeyev M, Shokolenko I, Wilson G, LeDoux S (2013). The maintenance of mitochondrial DNA integrity--critical analysis and update. Cold Spring Harb Perspect Biol.

[7] Smith DR. The past, present and future of mitochondrial genomics: have we sequenced enough mtDNAs? Brief Funct Genomics 2016; 15(1): 47–54. 10.1093/bfgp/elv027. 10.1093/bfgp/elv027 PMC4812591 26117139

[8] Pyron RA, Wiens JJ (2011). A large-scale phylogeny of Amphibia including over 2800 species, and a revised classification of extant frogs, salamanders, and caecilians. Mol Phylogenet Evol.

[9] Feng YJ, Blackburn DC, Liang D, Hillis DM, Wake DB, Cannatella DC, Zhang P (2017). Phylogenomics reveals rapid, simultaneous diversification of three major clades of Gondwanan frogs at the Cretaceous-Paleogene boundary. Proc Natl Acad Sci U S A.

[10] Lavrov DV, Pett W (2016). Animal mitochondrial dna as we do not know it: MT-genome organization and evolution in nonbilaterian lineages. Genome Biol Evol.

[11] Johnston IG, Williams BP (2016). Evolutionary inference across eukaryotes identifies specific pressures favoring mitochondrial gene retention. Cell Syst.

[12] Gray MW (2012). Mitochondrial evolution. Cold Spring Harb Perspect Biol.

[13] Adams KL, Palmer JD (2003). Evolution of mitochondrial gene content: gene loss and transfer to the nucleus. Mol Phylogenet Evol.

[14] Zhou X, Lin Q, Fang W, Chen X (2014). The complete mitochondrial genomes of sixteen ardeid birds revealing the evolutionary process of the gene rearrangements. BMC Genomics.

[15] Xia Y, Zheng Y, Miura I, Wong PB, Murphy RW, Zeng X (2014). The evolution of mitochondrial genomes in modern frogs (Neobatrachia): nonadaptive evolution of mitochondrial genome reorganization. BMC Genomics.

[16] Satoh TP, Miya M, Mabuchi K, Nishida M (2016). Structure and variation of the mitochondrial genome of fishes. BMC Genomics.

[17] Okajima Y, Kumazawa Y (2010). Mitochondrial genomes of acrodont lizards: timing of gene rearrangements and phylogenetic and biogeographic implications. BMC Evol Biol.

[18] Shapiro JA, von Sternberg R (2005). Why repetitive DNA is essential to genome function. Biol Rev Camb Philos Soc.

[19] D’Haeseleer P (2006). What are DNA sequence motifs?. Nat Biotechnol.

[20] Reams AB, Roth JR (2015). Mechanisms of gene duplication and amplification. Cold Spring Harb Perspect Biol.

[21] Qian Z, Adhya S (2017). DNA repeat sequences: diversity and versatility of functions. Curr Genet.

[22] Lopez-Flores I, Garrido-Ramos MA (2012). The repetitive DNA content of eukaryotic genomes. Genome Dyn.

[23] Shamanskiy VA, Timonina VN, Popadin KY, Gunbin KV (2019). ImtRDB: a database and software for mitochondrial imperfect interspersed repeats annotation. BMC Genomics.

[24] Ishino Y, Krupovic M, Forterre P (2018). History of crispr-cas from encounter with a mysterious repeated sequence to genome editing technology. J Bacteriol.

[25] Wells RD (2007). Non-B DNA Conformations, mutagenesis and disease. Trends Biochem Sci.

[26] Miesel L, Segall A, Roth JR (1994). Construction of chromosomal rearrangements in Salmonella by transduction: inversions of non-permissive segments are not lethal. Genetics.

[27] Bzymek M, Lovett ST (2001). Instability of repetitive DNA sequences: the role of replication in multiple mechanisms. Proc Natl Acad Sci U S A.

[28] Aras RA, Kang J, Tschumi AI, Harasaki Y, Blaser MJ (2003). Extensive repetitive DNA facilitates prokaryotic genome plasticity. Proc Natl Acad Sci U S A.

[29] Yang JN, Seluanov A, Gorbunova V (2013). Mitochondrial inverted repeats strongly correlate with lifespan: mtDNA inversions and aging. PLoS One.

[30] Solano A, Gamez J, Carod FJ, Pineda M, Playan A, Lopez-Gallardo E, Andreu AL, Montoya J (2003). Characterisation of repeat and palindrome elements in patients harbouring single deletions of mitochondrial DNA. J Med Genet.

[31] Lakshmanan L, Yee Z, Gruber J, Halliwell B, Gunawan R. Thermodynamic analysis of mitochondrial DNA breakpoints reveals mechanistic details of deletion mutagenesis. bioRxiv. 2018. 10.1101/254631.

[32] Lakshmanan L, Gruber J, Halliwell B, Gunawan R (2012). Role of direct repeat and stem-loop motifs in mtDNA deletions: cause or coincidence?. PLoS One.

[33] Chen T, He J, Huang Y, Zhao W (2011). The generation of mitochondrial DNA large-scale deletions in human cells. J Hum Genet.

[34] San Mauro D, Gower DJ, Zardoya R, Wilkinson M (2006). A hotspot of gene order rearrangement by tandem duplication and random loss in the vertebrate mitochondrial genome. Mol Biol Evol.

[35] Lerat E (2010). Identifying repeats and transposable elements in sequenced genomes: how to find your way through the dense forest of programs. Heredity.

[36] Hubley R, Finn RD, Clements J, Eddy SR, Jones TA, Bao W, Smit AF, Wheeler TJ (2016). The Dfam database of repetitive DNA families. Nucleic Acids Res.

[37] Bao W, Kojima KK, Kohany O (2015). Repbase update, a database of repetitive elements in eukaryotic genomes. Mob DNA.

[38] Sablok G, Padma Raju GV, Mudunuri SB, Prabha R, Singh DP, Baev V, Yahubyan G, Ralph PJ, La Porta N. ChloroMitoSSRDB 2.00: more genomes, more repeats, unifying SSRs search patterns and on-the-fly repeat detection. Database. 2015;2015. 10.1093/database/bav084. 10.1093/database/bav084 PMC4584093 26412851

[39] Kumar M, Kapil A, Shanker A (2014). MitoSatPlant: mitochondrial microsatellites database of viridiplantae. Mitochondrion.

[40] Goios A, Meirinhos J, Rocha R, Lopes R, Amorim A, Pereira L (2006). RepeatAround: a software tool for finding and visualizing repeats in circular genomes and its application to a human mtDNA database. Mitochondrion.

[41] Cechova J, Lysek J, Bartas M, Brazda V (2018). Complex analyses of inverted repeats in mitochondrial genomes revealed their importance and variability. Bioinformatics.

[42] Scheibye-Knudsen M, Fang EF, Croteau DL, Wilson DM, Bohr VA (2015). Protecting the mitochondrial powerhouse. Trends Cell Biol.

[43] Kauppila JH, Stewart JB. Mitochondrial DNA: Radically free of free-radical driven mutations. Biochim Biophys Acta 2015; 1847(11): 1354–1361. 10.1016/j.bbabio.2015.06.001. 10.1016/j.bbabio.2015.06.001 26050972

[44] Gissi C, Iannelli F, Pesole G (2008). Evolution of the mitochondrial genome of Metazoa as exemplified by comparison of congeneric species. Heredity.

[45] Letunic I, Bork P. Interactive tree of life (iTOL) v3: an online tool for the display and annotation of phylogenetic and other trees. Nucleic Acids Res 2016; 44(W1):W242–W245. 10.1093/nar/gkw290. 10.1093/nar/gkw290 PMC4987883 27095192

