# Supplementary material for: Repetitive DNA profile of the amphibian mitogenome
Source: BMC Bioinformatics. 2020 May 19;21:197. doi: 10.1186/s12859-020-3532-8 (PMC7236288; doi:10.1186/s12859-020-3532-8)
Supplement: Supplementary file 2 — Additional file 2: Figure S2. Empirical distributions of the Abundance of repeat sequences with lengths from 5 to 20 bp. [file 12859_2020_3532_MOESM2_ESM.pdf]

Repetitive DNA profile of the amphibian mitogenome

Noel Cabañas[1], Arturo Becerra[2], David Romero[3], Tzipe Govezensky[1],
Jesús Javier Espinosa-Aguirre[1], and Rafael Camacho-Carranza[1,2]*

[1]Instituto de Investigaciones Biomédicas, Universidad Nacional Autónoma de México, Cd. Universitaria, 04510, Cd. Mx., México, [2]Facultad de Ciencias, Universidad Nacional Autónoma de México, Cd. Universitaria, 04510, Cd. Mx., México, and [3]Centro de Ciencias Genómicas, Universidad Nacional Autónoma de México, Cuernavaca, Morelos, México. *To whom correspondence should be addressed. Contact: **rcamacho@biomedicas.unam.mx**

This file includes data that complement the information and enhance the interpretation of the main text.

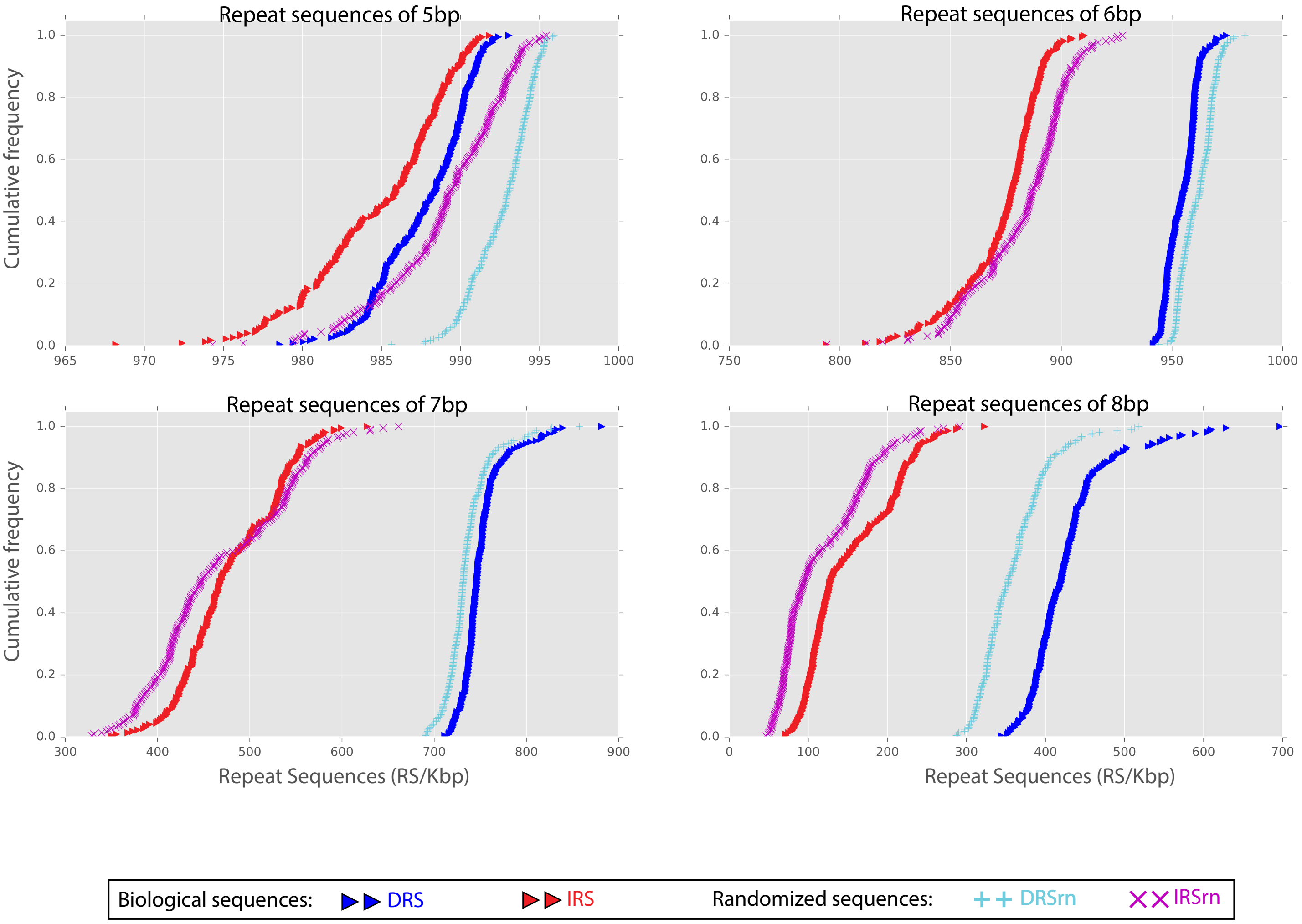

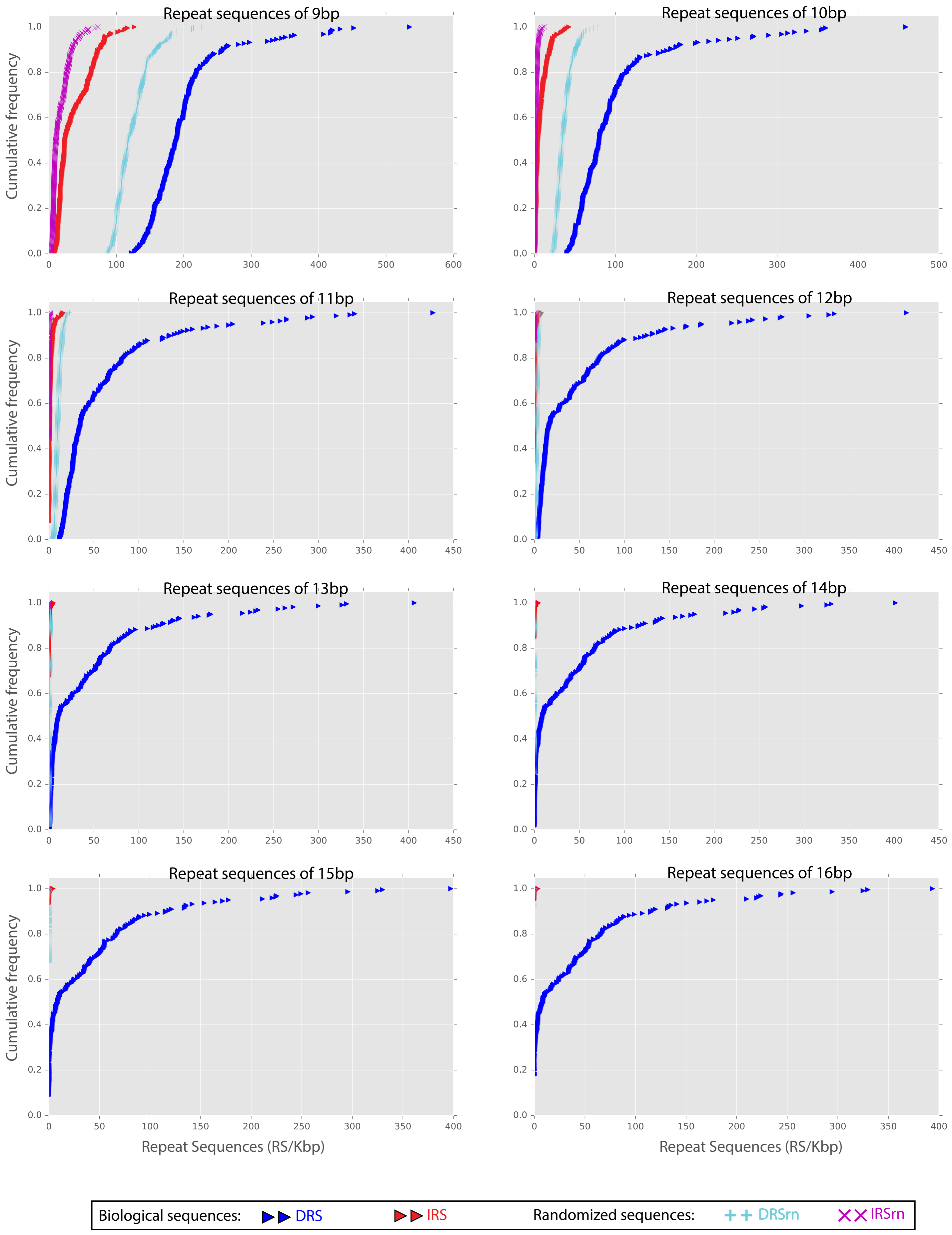

Repeat sequences of 9bp
Repeat sequences of 10bp
Repeat sequences of 11bp
Repeat sequences of 12bp
Repeat sequences of 13bp
Repeat sequences of 14bp
Repeat sequences of 15bp
Repeat sequences of 16bp
Cumulative frequency
Repeat Sequences (RS/Kbp)
Biological sequences: DRS IRS
Randomized sequences: DRSrn IRSrn

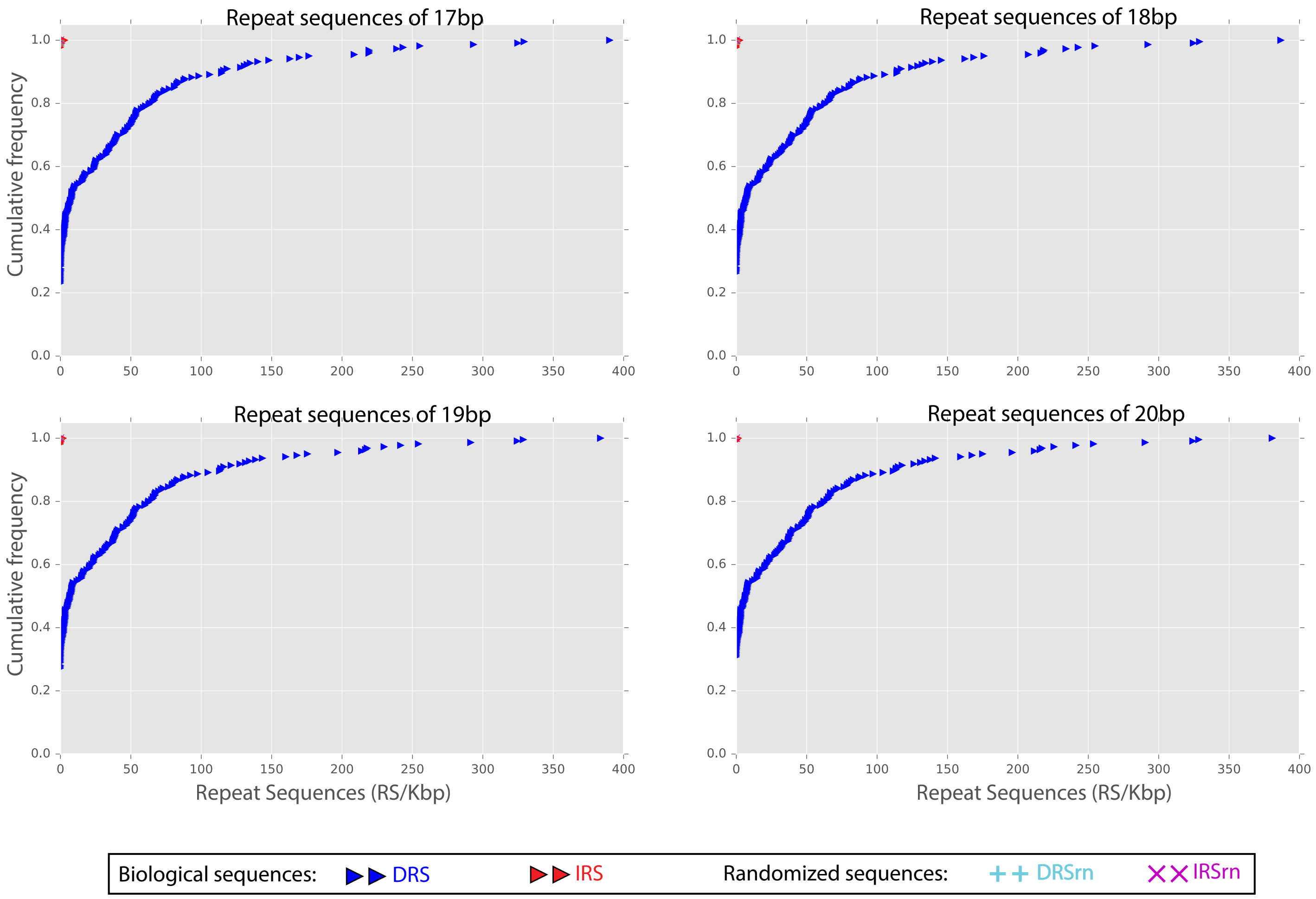

Additional files 2. Empirical distributions (EDF) of the abundance of repeat sequences with lengths from 5bp to 20 bp. These plots show the number of DRs and IRs per kilobase pairs in the 221 amphibian mitogenomes. We additionally show and compare the abundance of repeat sequences obtained from randomized genomes. Each figure presents an independent analysis for each specific size of repeat sequence. EDF with the number of repeat sequences from 21bp to 30bp were performed, but are not included in this supplementary file since the distributions are similar to the last ones displayed.
