# Supplementary material for: Repetitive DNA profile of the amphibian mitogenome
Source: BMC Bioinformatics. 2020 May 19;21:197. doi: 10.1186/s12859-020-3532-8 (PMC7236288; doi:10.1186/s12859-020-3532-8)
Supplement: Supplementary file 3 — Additional file 3: Figure S3. The Abundance of repeat sequences of 9 bp, 7 bp, 9 bp,15 bp in the amphibian phylogenetic tree. [file 12859_2020_3532_MOESM3_ESM.pdf]

Repetitive DNA profile of the amphibian mitogenome

Noel Cabañas[1], Arturo Becerra[2], David Romero[3], Tzipe Govezensky[1], Jesús Javier Espinosa-Aguirre[1], and Rafael Camacho-Carranza[1,2]*

[1]Instituto de Investigaciones Biomédicas, Universidad Nacional Autónoma de México, Cd. Universitaria, 04510, Cd. Mx., México, [2]Facultad de Ciencias, Universidad Nacional Autónoma de México, Cd. Universitaria, 04510, Cd. Mx., México, and [3]Centro de Ciencias Genómicas, Universidad Nacional Autónoma de México, Cuernavaca, Morelos, México. ***To whom correspondence should be addressed. Contact: **rcamacho@biomedicas.unam.mx**

These phylogenetic trees that display the abundance of repeat sequences in the amphibian mitogenomes and many others generated in this work can be visualized and exported from the iTOL webpage following this link: https://itol.embl.de/shared/salmonellaiib

Abundance of repeat sequneces of 5 bp

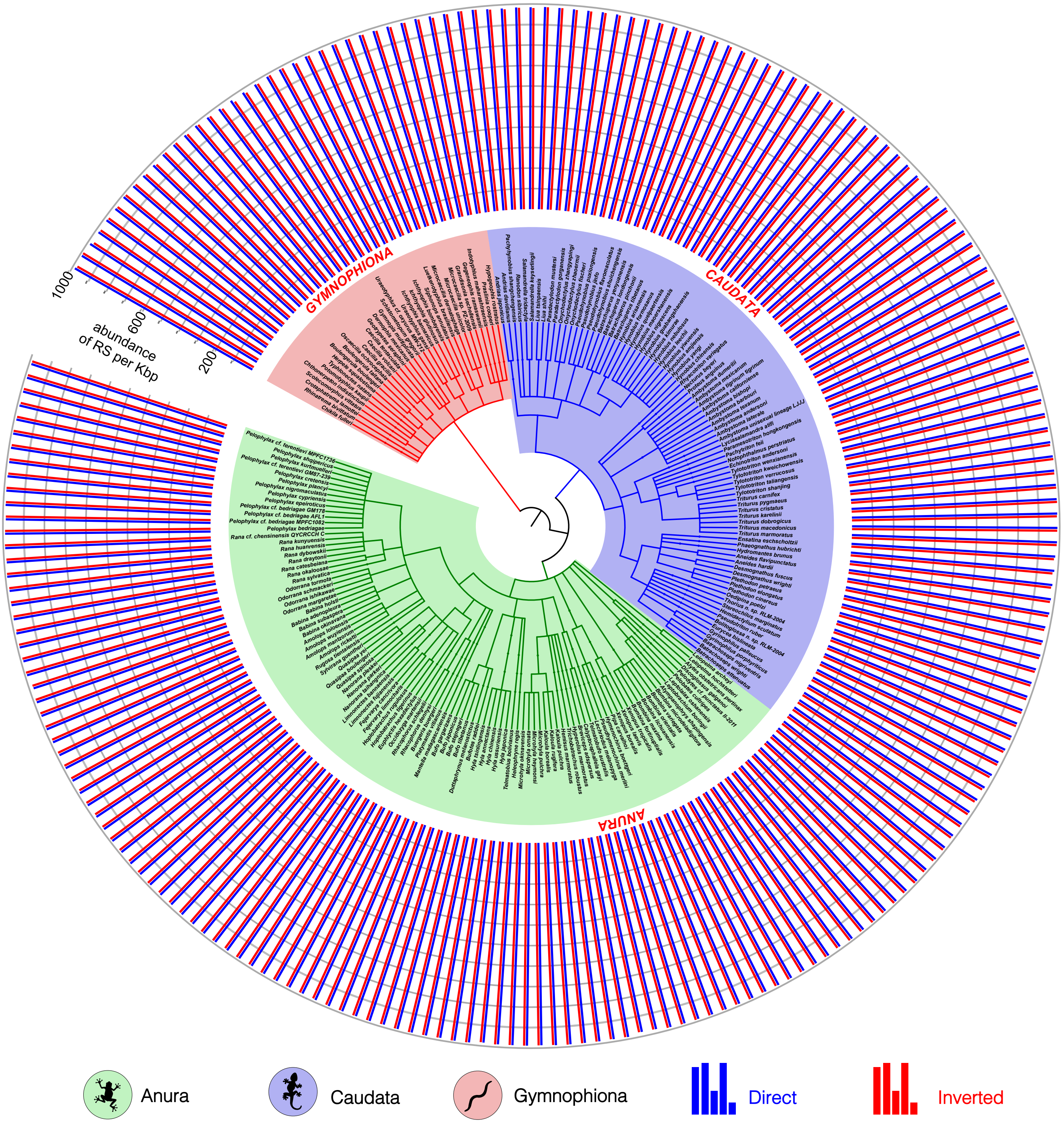

Abundance of repeat sequneces of 7 bp

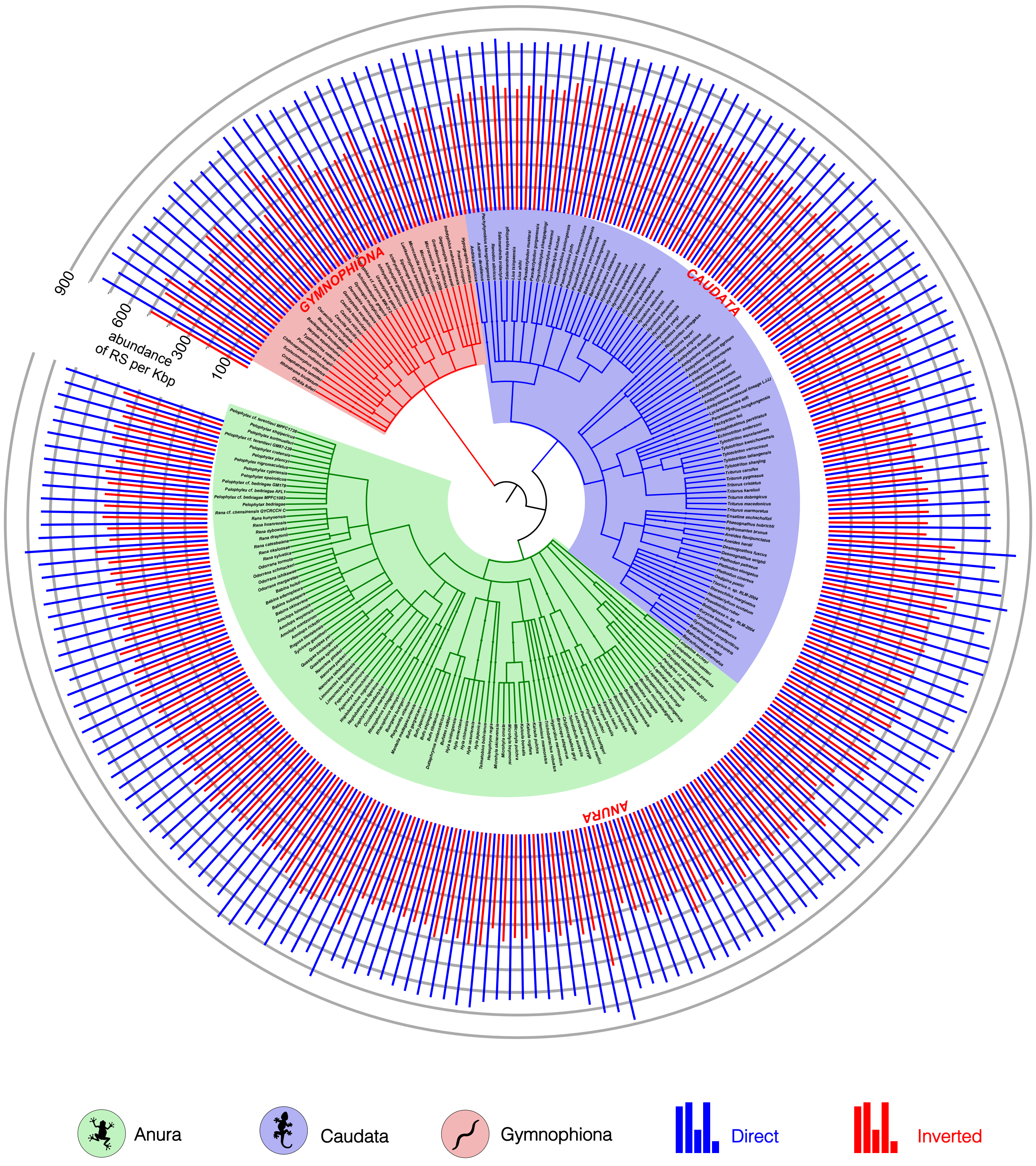

Abundance of repeat sequneces of 9 bp

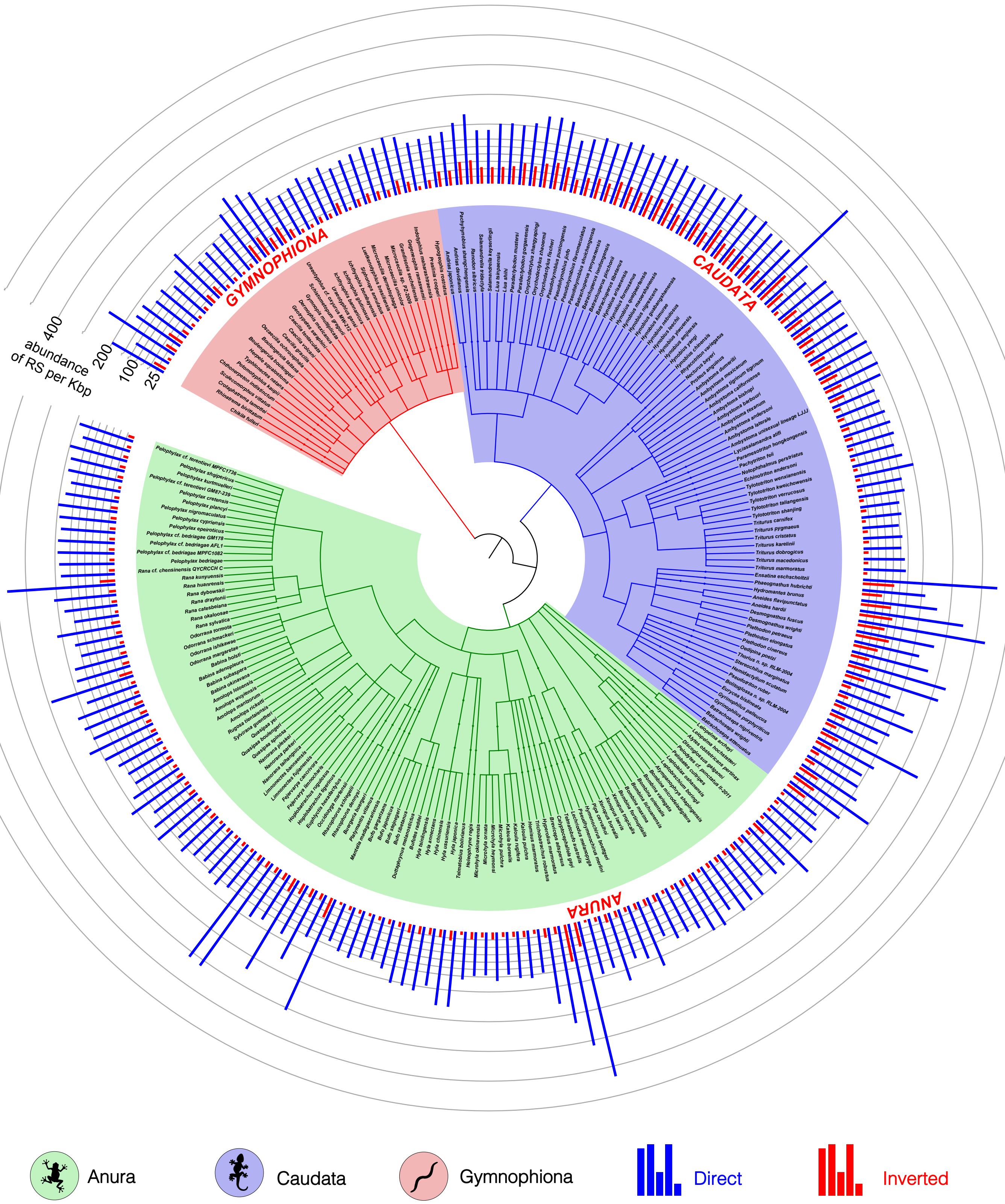

Abundance of repeat sequneces of 15 bp

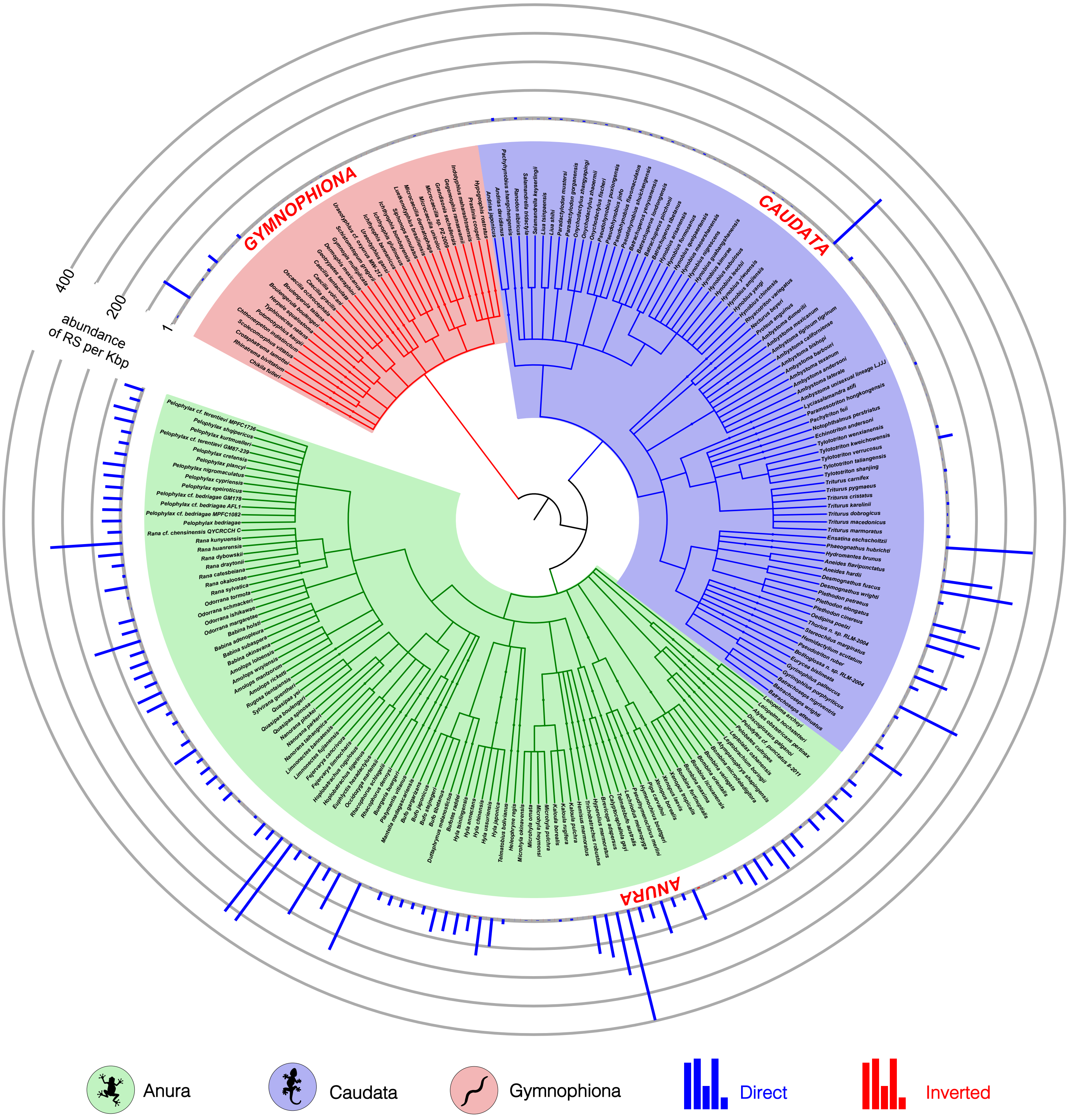

Additional files 3. The abundance of repeat sequences of 5 bp, 7 bp, 9bp, and 15bp in the amphibian phylogenetic tree. These plots present the 221 amphibians classified into three orders, with their respective abundance of direct (blue bars) and inverted (red bars) repeat sequences shown as bar graphs next to each other. The scale that shows the number of repeat sequences per kilobase pairs is represented with circular gray lines. Additional distributions of other sizes of repeat sequences can be accessed online within this link: https://itol.embl.de/shared/salmonellaiib
