# Supplementary material for: Repetitive DNA profile of the amphibian mitogenome
Source: BMC Bioinformatics. 2020 May 19;21:197. doi: 10.1186/s12859-020-3532-8 (PMC7236288; doi:10.1186/s12859-020-3532-8)
Supplement: Supplementary file 4 — Additional file 4: Figure S4. Bias of the direct and inverted repeat sequences in the amphibian mitogenomes with an atypical abundance of repeats. [file 12859_2020_3532_MOESM4_ESM.docx]

Repetitive DNA profile of the amphibian mitogenome

Noel Cabañas^1^, Arturo Becerra^2,^ David Romero^3^, Tzipe Govezensky^1^, Jesús Javier Espinosa-Aguirre^1^ and Rafael Camacho-Carranza^1,2*^

^1^Instituto de Investigaciones Biomédicas, Universidad Nacional Autónoma de México, Cd. Universitaria, 04510, Cd. Mx., México, ^2^Facultad de Ciencias, Universidad Nacional Autónoma de México, Cd. Universitaria, 04510, Cd. Mx., México, and ^3^Centro de Ciencias Genómicas, Universidad Nacional Autónoma de México, Cuernavaca, Morelos, México.

**Additional files 4a. Biases of the direct repeat sequences in the amphibian mitogenomes.** The distribution of direct repeats of 9 a bp and 30 bp in some mitogenomes. The direct repeats in the in the mitogenomes with an atypical abundance of repeat sequences are mainly distributed in non-genic regions (*Breviceps adpersus* and *Hoplobatrachus tigerinus*). This pattern is not followed by the amphibian mitogenomes with an average abundance of repeat sequences as the mitogenome of *Tylototriton verrucosus,* they display the repeat sequences all over the mitogenome without preferences *.*

**Supplementary Figures 4b. Biases of the inverted repeat sequences in the amphibian mitogenomes.** The distribution of inverted repeats of 9 a bp in some mitogenomes. The inverted repeats in the in the mitogenomes with an atypical abundance of repeat sequences are mainly distributed in genic regions (*Hyperolius marmoratus* and *Breviceps aspersus*). This pattern is not followed by the amphibian mitogenomes with an average abundance of repeat sequences as the mitogenome of *Ambystoma bishop* and *Dermophis mexicanus,* they display the repeat sequences all over the mitogenome without preferences*.*
