# Supplementary material for: Repetitive DNA profile of the amphibian mitogenome
Source: BMC Bioinformatics. 2020 May 19;21:197. doi: 10.1186/s12859-020-3532-8 (PMC7236288; doi:10.1186/s12859-020-3532-8)
Supplement: Supplementary file 5 — Additional file 5. Examples of the possible uses of the repeat sequences for identification and classification of amphibians. [file 12859_2020_3532_MOESM5_ESM.pdf]

Repetitive DNA profile of the amphibian mitogenome

Noel Cabañas[1], Arturo Becerra[2], David Romero[3], Tzipe Govezensky[1], Jesús Javier Espinosa-Aguirre[1] and Rafael Camacho-Carranza[1,2*]

[1]Instituto de Investigaciones Biomédicas, Universidad Nacional Autónoma de México, Cd. Universitaria, 04510, Cd. Mx., México, [2]Facultad de Ciencias, Universidad Nacional Autónoma de México, Cd. Universitaria, 04510, Cd. Mx., México, and [3]Centro de Ciencias Genómicas, Universidad Nacional Autónoma de México, Cuernavaca, Morelos, México.

Using the repeat sequences as a tool for Identification of the amphibians

We show an example of the possible use of repeat sequences as a tool for the identification and classification of amphibians. In figure **Additional files 5a,** we present the relation between direct repeats and inverted repeats in the members of the amphibian family Ambystomatidae that we included in our study. The results show that using a comparative method is possible to detect differences in the relation of repeat sequences, particularly in sizes between 9 to 14 for this family. These differences allow us to discern between the species of the family Ambystomatidae, thus can potentially be implemented as a way for the identification of organisms.

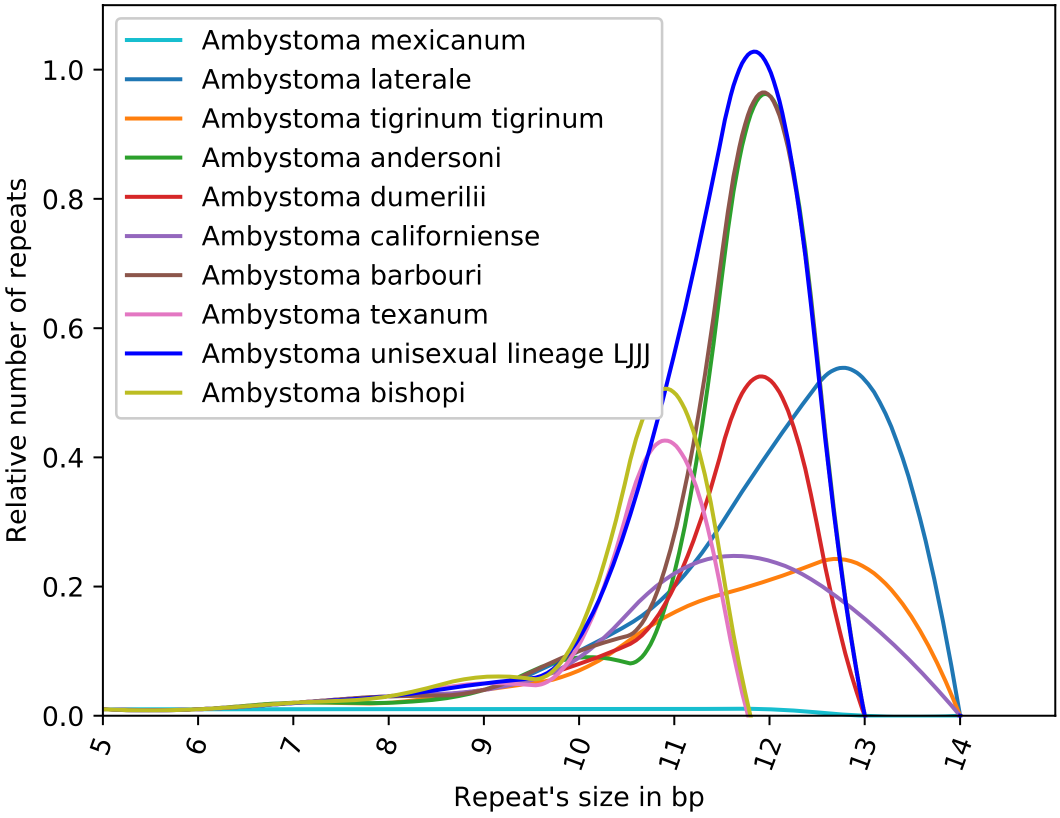

Additional files 5a. Differences in the relation of direct and inverted repeats in the Ambystomatidae family as an example of how it is possible to discern among species. The relation between direct and inverted shows that the repeat sequences can potentially be employed as a tool to discern among species. The x-axis shows a relative scale of the number of repeats. The y-axis shows the relation between direct and inverted repeats (direct repeats/inverted repeats).

Using the repeat sequences as a tool for classification of amphibians

Amphibians are organisms that present great variability in the size and structure of their genomes. Therefore, the classification of these organisms is still subject of debate [1]. Using as an example the Ambystomatidae family, we can notice that the information of the reference databases to obtain phylogenetic information about the amphibians (NCBI taxonomy, AmphibiaWeb, and The Tree of Life Web Project (http://tolweb.org/Ambystomatidae/15448)) display that the majority of Ambystoma species as almost identical, even when there is evidence that these organisms present an extremely flexible reproductive system [1, 2]. Some species are sexual, unisexual, or present different ploidy levels, which result in numerous biotypes [2, 3]. Phylogenetic studies trying to implement a taxonomic classification of the Ambystomatidae family have shown that even when using nuclear and mitochondrial markers, the phylogenies differ depending on the employed method [1, 4]. Here, we show how the identification of the repeat sequences can be implemented as a complementary method to resolve discrepancies in the amphibians' classification.

Examples of discrepancies in phylogenies of the family Ambystomatidae

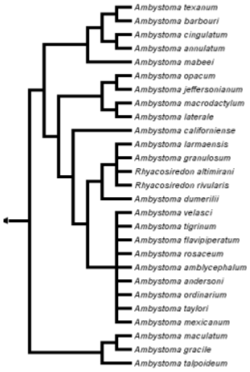

Ambystomatidae from The Tree of Life Web Project (ToL)
http://tolweb.org/tree/phylogeny.html

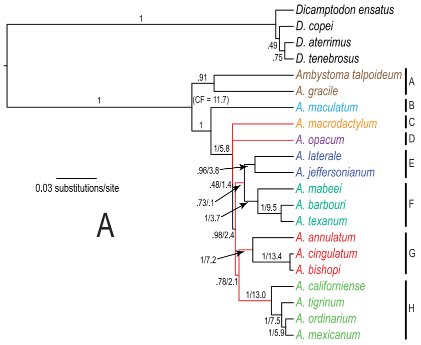

Ambystomatidae from Williams *et al.* 2013

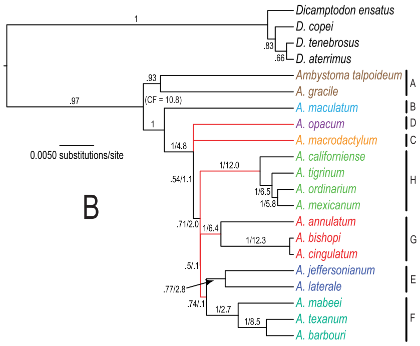

Ambystomatidae from Williams *et al.* 2013

We show in figure **Additional files 5b** how the relation of repeat sequences generates five different groups. Demonstrating that the mitogenomic structure, based on repeat sequences, is able to display relationships among organisms. For example, the genomes of *A. unisexual, A. Californiense*, and *A. laterale* display a greater similarity in the repeat sequences compared with the other members of the Ambystomatidae family. We can see as well that *A. bishopi* and *A. texanum* present a relation of repeat sequences nearly similar. It is worthwhile to mention that the majority of the classifications of the Ambystomatidae family place *A. mexicanum* near to *A. californiense,* while the relation of repeat sequences displays that *A. mexicanum* seems to be a special case in this family showing less variability in the number of repeats compared with their closer relatives, but if we construct a phylogenetic relationship *A. mexicanum* should be near to *A. dumerilii* and *A. barboury,* at least when using the relation of repeat sequences. The last comparison of *A. mexicanum* is congruent as well with ecological and geographic evidence. *A. mexicanum* is a species restricted to Mexico and presents a reduced population, for which we hypothesize limits the changes generated in its mitochondrial DNA. Our results presented in figure **Additional files 5b** support the phylogeny of Williams *et al.* 2013 that it is displayed with the letter B in the figure of examples of phylogenies. Using repeat sequences can serve as a complementary tool that can help with the classification at the level of families or orders rather than the creation of a global classification of organisms, but we need to take into account information as the distribution of repeat sequences, the abundance of repeat sequences with different sizes, or the comparison of the abundance of repeat sequences in the phylogenetic tree that can contribute to spot differences that lead to a classification. Here, we show an example only based on the relation of direct and inverted repeat sequences. However, we consider that the creation of profiles of repeat sequences rather than solely the number of repeat sequences is the information that should be used to create effective classifications. Otherwise, classification or identification based only on the number of repeat sequences could be limited, but not impossible as several authors have shown recently with the creation of phylogenies based in repeat sequences. [5, 6].

The differences in the relation of repeat and inverted sequences in the mitogenomes allow us to see differences to discern among species, and at the same time, we can obtain relationships, based in the mitochondrial repeat sequences, that can be used for classification.

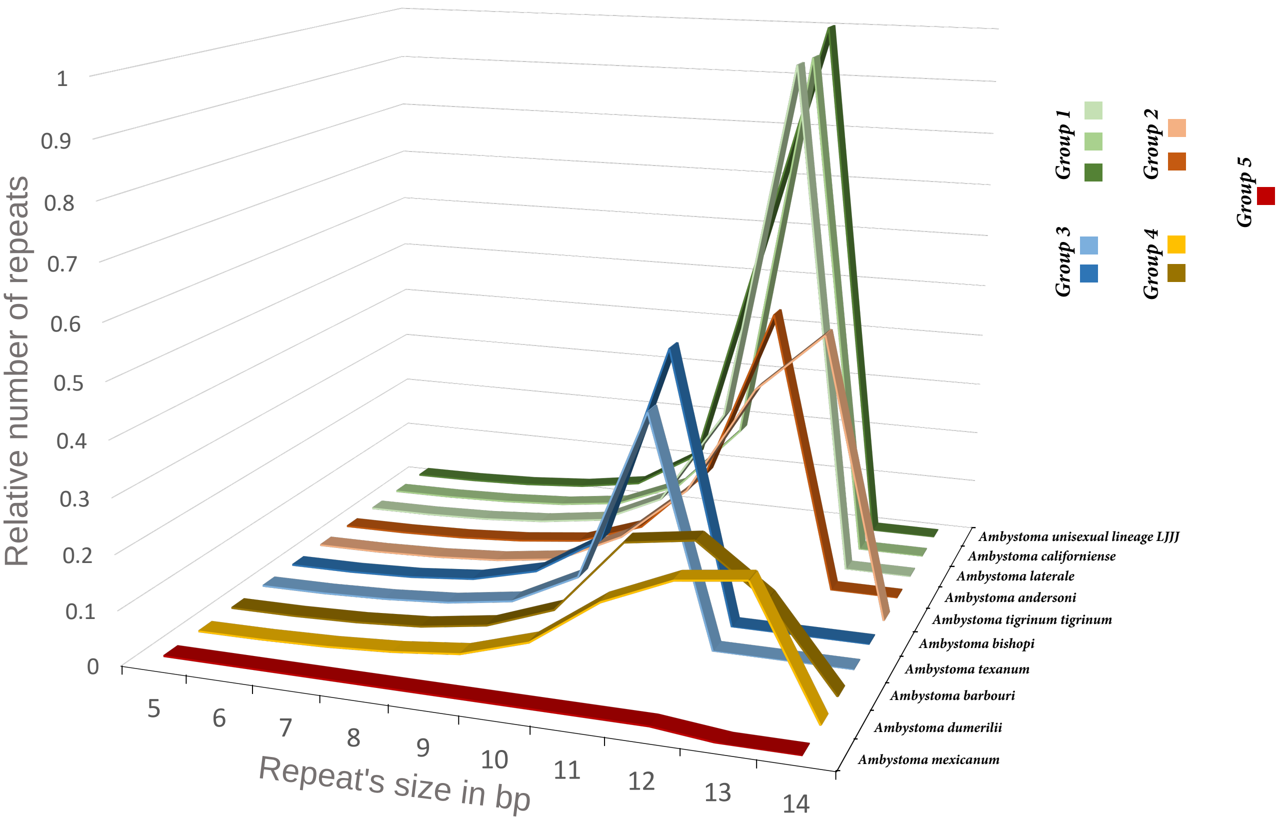

Additional files 5b. Resolving discrepancies in the classification of the Ambystomatidae family. Relation of direct and inverted repeat sequences (direct/inverted) showing how it is possible to group the Ambystomatidae family, which can help us to solve the discrepancies in their classification.

References

1. Pyron RA, Wiens JJ. A large-scale phylogeny of Amphibia including over 2800 species, and a revised classification of extant frogs, salamanders, and caecilians. *Mol Phylogenet Evol.* 2011; **61**(2):543-583. Available from: https://doi.org/10.1016/j.ympev.2011.06.012
2. Bi K, Bogart JP, Fu J. The prevalence of genome replacement in unisexual salamanders of the genus Ambystoma (Amphibia, Caudata) revealed by nuclear gene genealogy. *BMC Evol Biol.* 2008; **8**:158. Available from: https://doi.org/10.1186/1471-2148-8-158
3. Bogart JP, Bi K, Fu J, Noble DW, Niedzwiecki J. Unisexual salamanders (genus Ambystoma) present a new reproductive mode for eukaryotes. *Genome.* 2007; **50**(2):119-136. Available from: https://doi.org/10.1139/g06-152
4. Williams JS, Niedzwiecki JH, Weisrock DW. Species tree reconstruction of a poorly resolved clade of salamanders (Ambystomatidae) using multiple nuclear loci. *Mol Phylogenet Evol.* 2013; **68**(3):671-682. Available from: https://doi.org/10.1016/j.ympev.2013.04.013
5. Bolsheva NL, Melnikova NV, Kirov IV, Dmitriev AA, Krasnov GS, Amosova capital A C *et al*. Characterization of repeated DNA sequences in genomes of blue-flowered flax. *BMC Evol Biol.* 2019; **19**(Suppl 1):49. Available from: https://doi.org/10.1186/s12862-019-1375-6
6. Dodsworth S, Chase MW, Kelly LJ, Leitch IJ, Macas J, Novak P, Piednoel M, Weiss-Schneeweiss H, Leitch AR. Genomic repeat abundances contain phylogenetic signal. *Syst Biol.* 2015; **64**(1):112-126. Available from: https://doi.org/10.1093/sysbio/syu080
